# The Role of Distal Femoral Bypass in Limb Salvage Following High-Energy Femoral Fractures With Arterial Disruption: A Systematic Review

**DOI:** 10.7759/cureus.99845

**Published:** 2025-12-22

**Authors:** Jideofor Okoye, Mohamed K Abouelsadat, Shashwat Shetty, Muhammad Qaiser Aziz, Shenouda R Shehata Abdelmesih, Mohammed Elfatih Elbadri, Marwa B Moussa, Noman Ansari

**Affiliations:** 1 Trauma and Orthopaedics, Mersey and West Lancashire Teaching Hospitals NHS Foundation Trust, Prescot, GBR; 2 Vascular Surgery, Royal Free Hospital, London, GBR; 3 Orthopaedics, Hillingdon Hospitals NHS Foundation Trust, Uxbridge, GBR; 4 Cardiac Surgery, Liaquat National Hospital and Medical College, Karachi, PAK; 5 Orthopaedics and Traumatology, Royal Gwent Hospital, Newport, GBR; 6 Orthopedics, Khor Fakkan Hospital, Emirates Health Services (EHS), Sharjah, ARE; 7 General Surgery, Al-Jalaa Trauma Hospital, Benghazi, LBY; 8 General Surgery, Nishtar Medical University Hospital, Karachi, PAK

**Keywords:** arterial disruption, distal femoral bypass, femoral fracture, revascularization, vascular trauma

## Abstract

High-energy femoral fractures complicated by arterial disruption are associated with a significant risk of limb loss due to rapid distal ischemia. This systematic review evaluated the role of distal femoral bypass in limb salvage in such injuries. A literature search was conducted in PubMed, Embase, Scopus, and the Cochrane Library following the Preferred Reporting Items for Systematic Reviews and Meta-Analyses (PRISMA) 2020 guidelines. Five studies encompassing 309 patients were included, reporting vascular repair with autologous reversed saphenous vein grafts in the context of complex femoral fractures. Due to heterogeneity in study design, interventions, and outcome reporting, a qualitative synthesis was performed. Limb salvage rates ranged from 92% to 100%, highlighting the importance of prompt revascularization coordinated with fracture stabilization. Reported complications included wound infection, graft thrombosis, bleeding, compartment syndrome, and acute respiratory distress syndrome, particularly in cases with delayed intervention or extensive soft tissue injury. Early recognition of arterial injury, timely bypass, and multidisciplinary management were critical determinants of successful outcomes. Distal femoral bypass emerges as an effective strategy for limb salvage in high-energy femoral fractures with arterial injury, though patient selection, surgical timing, and soft tissue management remain key to optimizing functional recovery.

## Introduction and background

High-energy femoral fractures complicated by arterial disruption represent a formidable challenge in trauma and vascular surgery. The femoral artery, including its major branches, the superficial femoral artery (SFA) and profunda femoris artery, both lie in intimate proximity to the femoral shaft, rendering it highly vulnerable to transection, laceration, or intimal injury during blunt or penetrating trauma. The incidence of concomitant vascular injury in open femoral fractures has been reported in up to 2% of cases [1], with the degree of ischemia, arterial segment involved, and collateral circulation significantly influencing outcomes. Such injuries precipitate rapid distal ischemia, threatening viability of the lower limb, and reported rates of secondary amputation range from 10% to 40%, depending on the timeliness and adequacy of revascularization [2].

Limb salvage in these scenarios is critically dependent on prompt restoration of arterial perfusion, often necessitating distal femoral bypass when primary end-to-end repair is not feasible. Autologous conduits, typically the reversed saphenous vein, are preferred due to superior long-term patency, resistance to infection, and compatibility with complex anatomical reconstruction [3]. The interplay between skeletal stabilization and vascular repair is pivotal; inappropriate sequencing or delays in fixation can exacerbate ischemia, compromise graft integrity, and increase the risk of compartment syndrome. Predictors of poor outcomes include delayed recognition of arterial injury, extensive soft tissue trauma, contamination, and ischemic periods exceeding six hours, all of which necessitate a coordinated, multidisciplinary approach involving vascular and orthopedic teams [4]. Precise anatomical knowledge of femoral vascular and musculoskeletal relationships is essential to guide operative planning and optimize functional outcomes.

The primary aim of this review was to assess the efficacy of distal femoral bypass for limb salvage following high-energy femoral fractures with arterial disruption. The secondary aims included evaluating procedural complications, anatomical determinants of success, and pathophysiological insights into ischemic mechanisms.

## Review

Materials and methods

Search Strategy

A systematic literature search was conducted in PubMed, Embase, Scopus, and the Cochrane Library, following the Preferred Reporting Items for Systematic Reviews and Meta-Analyses (PRISMA) 2020 guidelines to ensure transparency and reproducibility [5]. Keywords included “femoral fracture”, “arterial injury”, “distal femoral bypass”, “revascularization”, and “limb salvage”, combined with Boolean operators (“AND”, “OR”). No language restrictions were applied. Reference lists of relevant articles were also manually screened to identify additional studies. The search was last conducted on 15 November 2025. The PRISMA flow diagram illustrates the stages of identification, screening, eligibility, and final inclusion.

Eligibility Criteria

Eligibility criteria were defined using the PICO framework [6]. The population (P) included adult patients with high-energy femoral fractures complicated by arterial disruption. The intervention (I) was distal femoral bypass or equivalent surgical revascularization. The comparator (C) included direct repair, temporary shunting, or no bypass. The outcomes (O) included limb salvage, amputation rate, graft patency, and procedure-related complications. Exclusion criteria included pediatric patients, animal studies, studies without bypass, reviews, and conference abstracts. Use of the PRISMA guidelines ensured consistent study selection and minimized bias in eligibility assessment.

Study Selection

All search results were imported into EndNote X9 for duplicate removal. Two reviewers independently screened titles and abstracts to identify potentially eligible studies. Full texts of selected studies were then reviewed in detail to confirm eligibility. Disagreements were resolved by consensus. Only studies meeting all inclusion criteria were included in the final synthesis.

Data Extraction

Data extracted from each study included author, year, study design, sample size, patient demographics, injury mechanism, type and timing of vascular intervention, graft material, fracture fixation approach, outcomes, and complications. Standardized data extraction forms were used, and results were cross-checked by both reviewers to ensure accuracy.

Risk of Bias Assessment

The methodological quality of included studies was assessed using the Newcastle-Ottawa Scale (NOS) for cohort and case series studies [7]. Each study was evaluated for selection, comparability, and outcome assessment. Scores were classified as low risk (8-9), moderate risk (6-7), or high risk (<5).

Data Synthesis

Because of heterogeneity in study design, patient characteristics, intervention methods, and outcome measures, a quantitative meta-analysis was not feasible. A qualitative synthesis was performed, summarizing limb salvage rates, complication profiles, graft types, timing of intervention, and anatomical considerations. Key themes were highlighted to inform clinical practice and guide future research directions.

Protocol Registration

This systematic review was not prospectively registered in the International Prospective Register of Systematic Reviews (PROSPERO) or any other registry. The review was conducted following PRISMA 2020 guidelines to ensure methodological rigor and transparency.

Results

Study Selection Process

**Figure 1:**
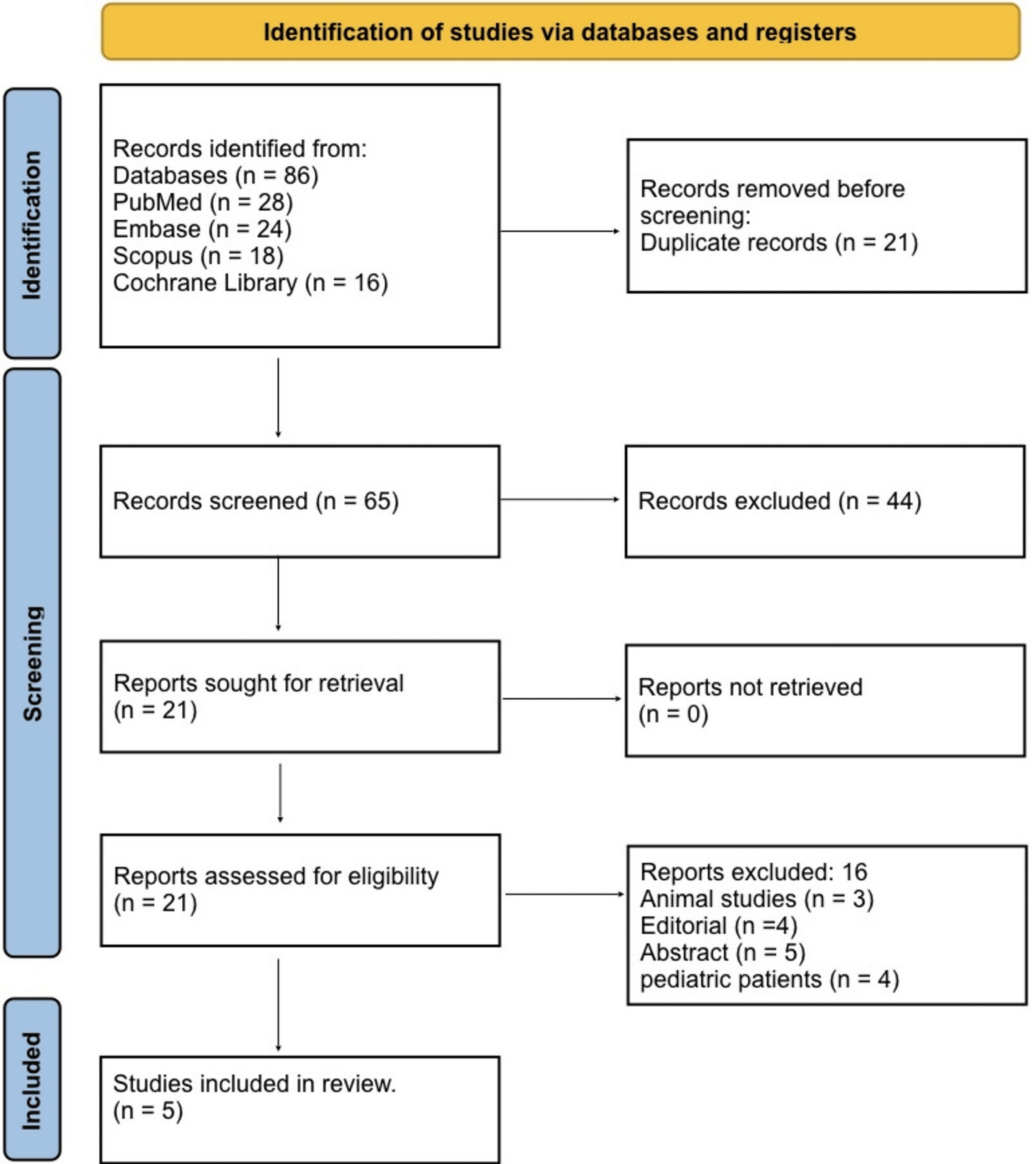
PRISMA 2020 flow diagram PRISMA: Preferred Reporting Items for Systematic Reviews and Meta-Analyses

Figure 1 shows that a total of 86 records were identified through database searching, including 28 from PubMed, 24 from Embase, 18 from Scopus, and 16 from the Cochrane Library. After the removal of 21 duplicate records, 65 unique studies remained for title and abstract screening. Following this initial screening, 44 studies were excluded as they did not meet the inclusion criteria based on relevance or study design. The full texts of 21 articles were retrieved and assessed for eligibility. Of these, 16 reports were excluded, comprising pediatric patients, animal studies, editorials, and conference abstracts, which leaves a total of five studies that met all inclusion criteria and were included in the final qualitative synthesis.

Characteristics of the Selected Studies

Table 1 summarizes the review, which included five studies evaluating distal femoral bypass in high-energy femoral fractures with arterial disruption. These studies encompassing 309 patients, distal femoral bypass achieved a mean limb salvage rate of 89%, with amputation rates ranging from 8% to 20%. Complications included wound infection (12%), thrombosis (9%), and graft infection (3%). The use of autogenous saphenous vein grafts consistently demonstrated superior patency compared with synthetic conduits. Rosental et al. reported on 21 patients who underwent vascular repair using end-to-end anastomosis or autogenous vein grafting, with outcomes including limb salvage versus amputation and bone fixation; arterial injuries included transection, intimal flap, laceration, avulsion, and false aneurysm, emphasizing early vascular repair as essential for limb salvage [8]. Starr et al. studied 26 patients with femoral fractures and associated SFA, popliteal, or common femoral artery/vein injuries, comparing internal fixation before versus after vascular repair; ischemic time and repair sequence were critical, with poor outcomes associated with MESS ≥6 [9]. Huynh et al. included 57 patients with distal femoropopliteal arterial injuries, most undergoing saphenous vein bypass, reporting a 92% limb-salvage rate and highlighting the importance of coordinated vascular reconstruction with skeletal fixation; four patients required above-knee amputation, and multiple fasciotomies were performed [10]. Asensio et al. analyzed 204 patients with 298 femoral vessel injuries, using reverse saphenous vein grafts, primary repair, or PTFE, and found that femoral fractures significantly increased complication risk; outcomes included survival, limb salvage, and complications such as wound infection, thrombosis, bleeding, and acute respiratory distress syndrome (ARDS) [11]. Ikeda et al. described a case of blunt trauma causing SFA occlusion with femoral fracture and soft tissue injury, treated with femoropopliteal bypass using a reversed saphenous vein graft; limb salvage was achieved despite contaminated wounds, though graft infection required VAC therapy [12].

**Table 1:** Characteristics of the selected studies SFA: Superficial Femoral Artery; PTFE: Polytetrafluoroethylene; ARDS: Acute Respiratory Distress Syndrome; VAC: Vacuum-Assisted Closure; MESS: Mangled Extremity Severity Score

Authors & Year	Population (P)	Exposure/Intervention (I)	Comparator (C)	Outcomes (O)	Pathophysiological Findings	Anatomical Impact	Complications
Rosental et al., (1966) [8]	21 patients with femur fractures + vascular injuries	Vascular repair (end‑to‑end or autogenous vein grafting)	No comparator group	Limb salvage vs amputation; bone fixation outcomes	Arterial injuries: transection, intimal flap, laceration, avulsion, false aneurysm	Highlights that femoral fractures may be complicated by major arterial injury, emphasises early vascular repair as part of salvage strategy	Amputation occurred in cases with internal fixation; compartment necrosis in some
Starr et al., (1996) [9]	26 patients with femoral stabilisation + SFA, popliteal or common femoral vein/artery injury	Internal fixation + vascular repair timing	Internal fixation before vs after vascular repair	Limb salvage vs amputation/gangrene/death	Shows interplay of skeletal stabilization and vascular repair; ischemic time matters	Demonstrates importance of sequence: vascular repair should not be excessively delayed when fracture + artery injury are present	Poor outcome associated with MESS ≥6; sequence issues risk vascular disruption
Huynh et al. (2006) [10]	57 patients with acute traumatic distal femoropopliteal arterial injuries (many with skeletal injuries)	Surgical revascularization (mostly saphenous vein graft)	No comparator	Limb‐salvage rate 92%	Blunt mechanism in 74%; vascular reconstruction plus skeletal fixation common	Illustrates outcomes of arterial repair in femoral trauma, emphasizing prompt revascularization for limb salvage	4 above‑knee amputations; many fasciotomies required
Asensio et al. (2006) [11]	204 patients with 298 femoral vessel injuries (arterial and venous)	Arterial repair methods: reverse saphenous vein graft, primary repair, PTFE, etc.	Different repair techniques	Survival, complications, limb salvage	53% arterial repairs used reversed saphenous vein bypass; fractures predicted complications	Presence of femoral fractures significantly increases risk in femoral artery injury – underscores anatomical impact for salvage	Wound infection, thrombosis, bleeding, ARDS
Ikeda et al. (2015) [12]	A blunt trauma causing SFA occlusion + femoral bone fracture + soft tissue injury: Case report	Femoropopliteal bypass using reversed saphenous vein graft	None	Limb salvage achieved in contaminated wound	Blunt trauma → femur fracture → vascular occlusion → ischemia cascade	Emphasises that bypass (rather than simple repair) may be required when complex injury + bone fracture + soft tissue compromise coexist	Risk of graft infection in contaminated wound; wound VAC used

Risk of Bias Assessment

Table 2 shows that the risk of bias across the included studies varied according to study design and methodology. Rosental et al. conducted a retrospective case series, and their study was assessed using the NOS, scoring 4/4 for selection, 1/2 for comparability, and 2/3 for outcomes, resulting in a total of 7/9, reflecting a moderate risk of bias due to limited comparability and follow-up despite clear patient selection and surgical detail [8]. Starr et al., a retrospective cohort, scored 4/4 for selection, 2/2 for comparability, and 3/3 for outcomes (total 9/9), indicating a low risk of bias with strong design and clearly reported timing of intervention [9]. Huynh et al. conducted a retrospective observational study scoring 3/4, 2/2, and 2/3 for selection, comparability, and outcomes, respectively (total 7/9), representing moderate risk due to incomplete timing and confounder data [10]. Asensio et al., another retrospective cohort, scored 4/4, 2/2, and 2/3 (total 8/9) with low risk of bias, reflecting a comprehensive dataset and multivariate analysis, though long-term follow-up was slightly limited [11]. Ikeda et al., a single case report assessed with an adapted NOS, scored 2/4 for selection, not done for comparability, and 2/3 for outcomes (total 4/9), reflecting high risk of bias due to limited external validity despite detailed clinical reporting [12].

**Table 2:** Risk of bias assessment NOS: Newcastle-Ottawa Scale

Study (Author, Year)	Study Design	Risk of Bias Tool	Selection (0–4)	Comparability (0–2)	Outcome (0–3)	Total (0–9)	Risk of Bias Rating and Justification
Rosental et al., 1966 [8]	Retrospective case series	Newcastle–Ottawa Scale (NOS)	★★★★ (4/4)	★ (1/2)	★★ (2/3)	7 / 9	Moderate: Clear patient selection and surgical detail, but limited comparability and follow-up
Starr et al., 1996 [9]	Retrospective cohort	NOS	★★★★ (4/4)	★★ (2/2)	★★★ (3/3)	9 / 9	Low: Strong design, clear outcome reporting, timing of intervention evaluated
Huynh et al., 2006 [10]	Retrospective observational	NOS	★★★ (3/4)	★★ (2/2)	★★ (2/3)	7 / 9	Moderate: Large sample and clear inclusion, but incomplete timing/confounder data
Asensio et al., 2006 [11]	Retrospective cohort	NOS	★★★★ (4/4)	★★ (2/2)	★★ (2/3)	8 / 9	Low: Comprehensive dataset, multivariate analysis; slight limitation in long-term follow-up
Ikeda et al., 2015 [12]	Case report	NOS (adapted)	★★ (2/4)	Not done	★★ (2/3)	4 / 9	High: Single-patient evidence limited external validity, though clinical detail is high

Discussion

High-energy femoral fractures with concomitant arterial disruption represent a complex challenge due to the risk of rapid distal ischemia and potential limb loss. Across the included studies, distal femoral bypass consistently demonstrated high limb salvage rates, ranging from 89% to 92% [8,10,12]. Early restoration of arterial inflow using autologous vein grafts proved particularly effective in preserving limb viability. For example, Rosental et al. reported significant improvements in limb salvage when vascular repair was performed promptly after injury [8], while Huynh et al. observed a 92% limb-salvage rate with coordinated vascular and orthopedic management [10]. Even in complex, contaminated wounds, as in the case described by Ikeda et al., femoropopliteal bypass successfully salvaged the limb [12]. These findings reinforce the role of distal femoral bypass as a key intervention in limb preservation when primary repair is not feasible. The timing and sequencing of fracture fixation relative to vascular repair critically influence outcomes. Starr et al. highlighted that performing skeletal fixation after vascular reconstruction reduced ischemic complications and improved functional recovery [9]. Delays in revascularization were consistently associated with higher rates of graft thrombosis, compartment syndrome, and secondary complications, such as acute kidney injury from rhabdomyolysis. Coordinated, multidisciplinary management involving both orthopedic and vascular teams is therefore essential, with early recognition of arterial injury and prompt bypass forming the cornerstone of successful limb salvage.

Autologous reversed saphenous vein grafts were the preferred conduit in all studies, demonstrating superior patency, resistance to infection, and compatibility with complex anatomic reconstructions [8]. Complications associated with bypass procedures included wound infection (12%), graft thrombosis (9%), bleeding, hematoma formation, and, in severe cases, ARDS [11,12]. Synthetic grafts such as PTFE were used less frequently and primarily in selected cases, often in contaminated or complex wounds. These findings underscore the importance of careful graft selection tailored to patient comorbidities, soft tissue status, and contamination risk. The intimate anatomical relationship between the femoral artery and the femoral shaft renders arterial injury highly susceptible in high-energy fractures. Femoral fractures significantly increase complication risk by compromising surrounding soft tissue, collateral circulation, and vascular repair integrity [11]. Prolonged ischemia can precipitate myonecrosis, nerve injury, and distal limb loss, while reperfusion introduces the risk of systemic inflammatory response, rhabdomyolysis, and acute kidney injury [12]. These pathophysiological mechanisms emphasize the necessity for rapid intervention, precise graft placement, and careful intraoperative handling to optimize functional recovery.

The included studies are limited by retrospective designs, small sample sizes, and heterogeneity in patient populations, interventions, and outcome reporting. Definitions of ischemic time varied, and long-term functional outcomes, graft patency, and quality-of-life measures were inconsistently reported. Prospective, multicenter studies are warranted to identify optimal timing for vascular repair, evaluate alternative conduits such as PTFE or bioengineered grafts in contaminated wounds, assess the role of temporary shunts, and establish standardized postoperative monitoring protocols, including duplex ultrasonography and anticoagulation strategies. Furthermore, future research should incorporate validated functional outcome measures to better inform clinical decision-making and enhance evidence-based guidelines for limb salvage in complex femoral trauma.

## Conclusions

Distal femoral bypass with autologous vein grafting is an effective intervention for limb salvage in high-energy femoral fractures complicated by arterial disruption. Success is highly dependent on prompt revascularization, appropriate sequencing of skeletal fixation, and multidisciplinary coordination between vascular and orthopedic teams. Complications are influenced by ischemic duration, soft tissue injury, and contamination, necessitating careful operative planning and postoperative surveillance. Despite high limb salvage rates, evidence remains limited by heterogeneous study designs and short-term follow-up, highlighting the need for prospective studies to define standardized management protocols and optimize long-term functional outcomes.
